# Feasibility of Dual-Task Gait Training for Community-Dwelling Adults after Stroke: A Case Series

**DOI:** 10.1155/2014/538602

**Published:** 2014-04-09

**Authors:** Prudence Plummer, Raymond M. Villalobos, Moira S. Vayda, Myriam Moser, Erin Johnson

**Affiliations:** ^1^Division of Physical Therapy, Department of Allied Health Sciences, The University of North Carolina at Chapel Hill, 3020 Bondurant Hall, Campus Box No. 7135, Chapel Hill, NC 27599, USA; ^2^New England Rehabilitation Hospital, Woburn, MA 01801, USA

## Abstract

This case series explored the feasibility and efficacy of cognitive-motor dual-task gait training in community-dwelling adults within 12 months of stroke. A secondary aim was to assess transfer of training to different dual-task combinations. Seven male participants within 12 months of stroke participated in 12 sessions of dual-task gait training. We examined single and dual-task performance in four different dual-task combinations at baseline, after 6 and 12 sessions, and if possible, at 1-month followup. Feasibility was assessed by asking participants to rate mental and physical fatigue, perceived difficulty, anxiety, and fear of falling at the end of each session. Five of the seven participants demonstrated reduced dual-task cost in gait speed in at least one of the dual-task combinations after the intervention. Analysis of the patterns of interference in the gait and cognitive tasks suggested that the way in which the participants allocated their attention between the simultaneous tasks differed across tasks and, in many participants, changed over time. Dual-task gait training is safe and feasible within the first 12 months after stroke, and may improve dual-task walking speed. Individuals with a combination of physical and cognitive impairments may not be appropriate for dual-task gait training.

## 1. Introduction

Cognitive-motor dual-task interference, defined as the decrement in performance that occurs when cognitive and motor tasks are performed simultaneously, has been well established in people after stroke [1–9]. This growing body of research has demonstrated significant dual-task decrements in gait speed [1, 5, 8], stride length [5, 8], cadence [6, 8], stride duration [2, 4, 8], and double limb support duration [1, 7]. In other words, compared to single-task walking, when individuals with stroke perform a cognitive task while walking they are less stable and take shorter, slower steps, resulting in a dramatic cost on gait speed. Gait-related dual-task deficits persist in community-dwelling stroke survivors many months after discharge from rehabilitation [3, 5, 8]. Since walking in the community is often performed concurrently with cognitive tasks, such as remembering directions or engaging in social interactions, a reduced capacity for dual-task walking may restrict the degree to which a person is able to physically function and participate in their life roles.

Conventional rehabilitation does not appear to adequately address gait-related dual-task interference. For example, in a longitudinal study of cognitive-motor interference, Cockburn and colleagues [2] found that 7 out of 10 patients showed a reduction in gait decrement associated with dual-task walking after usual rehabilitation; however, most patients continued to exhibit considerable dual-task interference during walking at discharge. Thus, even though single-task gait speed may recover to normal values after rehabilitation for stroke, dual-task capacity can remain considerably impaired.

There is promising evidence for dual-task training in older adults [10, 11] and individuals with Parkinson's disease [12, 13]. A recent study examined the effects of a cognitive-motor dual-task gait training intervention in people with neurological disorders [14], but only 2 of the 10 participants in the experimental group had experienced a stroke. To date, the only published dual-task training study in stroke is a dual-task exercise program involving patients with chronic stroke who walked while manipulating either one or two balls of various sizes (i.e., a motor-motor dual-task) [15]. Training was provided for 30 minutes, 3 times per week for 4 weeks. Compared to 12 patients who did not receive any intervention, the 13 patients who received dual-task training significantly improved their gait speed, cadence, stride duration, and stride length in single and dual-task (tray carrying) walking. Because this study focused on the coordination of simultaneous motor tasks and did not include any follow up, it is not known whether improvements transferred to other types of dual-tasks, such as cognitive-motor dual-tasks, or whether the improvements were maintained.

It is now well recognized that dual-task interference is influenced by the nature and difficulty of the cognitive tasks [9]. In a classic example, Maylor and Wing [16] found that age-related differences in postural stability were increased by cognitive tasks involving visuospatial cognition but not by counting tasks. Similar findings have been reported in gait-related dual-task interference in people with stroke [8]. Although these findings could be due to differences in task complexity, it is possible that the distinct cognitive processing demands of the tasks interfere differentially with postural control or gait, especially since gait is multifaceted with regard to its underlying cortical control mechanisms [17]. For this reason, it could be hypothesized that dual-task training in one type of task (e.g., executive function task) would not transfer to another type of cognitive task (e.g., visuospatial cognition task). Knowing whether the effects of dual-task training transfer to untrained dual-task combinations is essential for designing and planning rehabilitation interventions.

The purposes of this case series were to explore the feasibility and efficacy of a cognitive-motor dual-task training paradigm in community-dwelling adults within 12 months of stroke and to explore transfer of training to different dual-task combinations. To gain insight into whether dual-task gait training transfers across dual-task combinations, we examined the effects of the intervention on three different cognitive-motor dual-tasks and, where feasible, one motor-motor dual-task.

## 2. Case Descriptions

The case series included participants within 12 months of stroke who had completed conventional rehabilitation and were living in the community. Participants had to be able to walk at least 10 meters without the assistance of another person, follow a three-step command, and communicate verbally in English. Individuals were not eligible to participate if they had any pre-existing neurological disorders other than stroke, a previous stroke with residual deficits, uncorrected hearing impairment, severe visual impairment, severe dysarthria or aphasia, lower extremity amputation, any orthopedic problem affecting gait, concurrent participation in a trial of locomotor, or cognitive rehabilitation or were not living in the community prior to their stroke. Participants were screened for eligibility and approved for participation by a physician. Seven participants were recruited and provided written informed consent to participate.

## 3. Procedures

Study procedures were approved by the Institutional Review Board at Northeastern University (Boston, MA). Following baseline testing, participants completed 12 sessions of dual-task gait training (DTGT), 30 minutes each session, 3 times per week for 4 weeks. Maximum time to complete all sessions was 6 weeks. A midpoint assessment was conducted after 6 sessions. Posttraining assessments were completed within one week following the last session. If possible, a follow-up assessment 1 month later was also conducted. All training sessions and assessments took place in the outpatient therapy department at New England Rehabilitation Hospital (Woburn, MA). Licensed physical therapists were trained to provide the study intervention, and a trained research assistant conducted all of the outcome assessments.

## 4. Intervention

DTGT consisted of gait activities performed simultaneously with cognitive tasks. Gait training activities were based on Gentile's taxonomy of tasks [18]; the progressive sequence of gait training activities is presented in Table 1. Five categories of cognitive tasks with different levels of difficulty were used for the dual-task training (Table 2). The cognitive tasks used for DTGT were adapted from tasks used in previous dual-task training studies [10–12] and were selected to represent a range in level of difficulty. Tasks involving generation of spontaneous speech (e.g., telling a story) and visuospatial cognition (e.g., visualizing and reciting directions) were purposely excluded from the intervention regimen so that transfer of training to untrained tasks, such as these, could be assessed. The participants performed at least two different cognitive tasks in each training session.

Cognitive tasks were progressed according to individual abilities and progression occurred in concert with the progression of gait activities. The cognitive aspect of DTGT could be progressed in two ways: level of difficulty within the task (e.g., reciting a longer list of items from memory) or the category of task, recognizing that perceived task difficulty may vary between participants. For example, someone who is “good with numbers” might find the arithmetic-based tasks less difficult than someone who is not strong in arithmetic. Gait activities could be progressed by increasing speed, increasing the duration of continuous walking bouts, and changing the category/nature of the task (i.e., closed task versus open task). Therapists were instructed to ensure training activities were challenging, but not impossible, and were encouraged to try to progress training in both cognitive and gait domains within each session. However, several factors concerning the interaction between gait and cognitive tasks needed to be considered when progressing intervention activities. For example, walking speed may need to be decreased initially when a cognitive task is made more difficult; or an obstacle course activity may need to be made easier (e.g., closed task activity instead of variable activity) when a more difficult cognitive task is first attempted.

Therapists were provided with a comprehensive Manual of Procedures for the intervention and attended two 3-hour training sessions. The manual described each cognitive task and the progressive gait activities, as well as guidelines for progression of training. Therapists documented the duration, intensity, and type of treatment activities for each session on a standardized form. The importance of adhering strictly to the study protocol for the duration of the study was emphasized. The goal for all participants was to improve their ability to walk (increase speed, stride length, symmetry) while concurrently performing the cognitive tasks.

## 5. Outcome Measures

Gait and cognitive performances were assessed under single-task conditions and three different cognitive-motor dual-task conditions. Where feasible, we also assessed performance in a motor-motor dual-task. The three cognitive tasks were auditory Stroop, clock task, and spontaneous speech; the secondary motor task was a coin transfer task. The cognitive tasks were selected to represent different cognitive domains, one of which more closely resembled the cognitive activities used in training, while the others were considered “untrained” dual-task combinations (Table 3).

In the auditory Stroop task [19] participants heard the words “high” and “low” spoken in either a high pitch (360 Hz) or a low pitch (180 Hz). The participants were instructed to indicate the pitch of the word they heard (ignoring the actual word presented) by responding “high” or “low” as quickly and as accurately as possible. In the clock task, the participants heard a time (e.g., ten-twenty-six) and were required to respond “yes” if the hands were in same half (left/right) and “no” if they were not. For the spontaneous speech task, speech samples were elicited using a set of questions designed to stimulate a verbal response lasting at least 30 seconds (e.g., tell me what you did on the weekend).

The coin transfer task, adapted from previous research [20, 21], involved participants transferring US 25-cent coins, one at a time using the dominant hand, from the dominant-side “pocket” to the nondominant side as quickly as possible. The pockets (16.5 × 14.5 cm) were attached to a belt worn around the participant's waist. In all dual-task conditions, the participants were not specifically instructed to prioritize either task because we were primarily interested in observing the spontaneous dual-task effects. Performance in both gait and nongait tasks was assessed in order to make inferences about attention prioritization and patterns of cognitive-motor interference during dual-task conditions [9].

Spatiotemporal metrics of gait were assessed using a 6-meter GAITRite walkway, with a 2-meter runoff at each end to allow deceleration and turning. Participants completed 4–6 continuous passes for each task. The average of all GAITRite passes for each condition was used for analysis. Order of the dual-tasks (Stroop, clock, speech, and coin transfer) was randomized for each participant, but each person completed the tasks in the same order on each testing occasion. In this report, we focus on gait speed as the primary measure of gait performance due to its functional importance [22] and to enable comparison of findings to previous dual-task studies.

Single-task performance on the Stroop, clock, speech, and coin transfer tasks was performed immediately prior to the respective dual-task condition. Single-task performance for the coin transfer task was assessed whilst standing; for the cognitive tasks, single-task performance was assessed while sitting. Average reaction times (in milliseconds) and overall accuracy (percent correct) on the auditory Stroop and clock tasks were measured using DirectRT software (Empirisoft, New York, NY). Discourse analysis of the speech transcripts was performed to evaluate dual-task effects on speech. We focused on two measures of speech discourse: clauses per utterance, a measure of sentence complexity, and pauses per utterance. This decision was based on our previous research, which demonstrated that individuals with stroke experienced a significant dual-task effect on pauses per utterance but not on clauses per utterance [8]. In the coin transfer task, the number of coins transferred (max. 12) was recorded, and the coin transfer rate (coins/min) was calculated. In the single-task condition of the coin-transfer task, the participants transferred as many coins as possible in 30 seconds.

To examine the effects of the intervention on the amount of dual-task interference, we calculated dual-task effects (DTE) on gait speed and each secondary-task measure (i.e., reaction time, accuracy, speech variables, and coin transfer rate). The DTE represents the relative change in performance in the dual-task condition compared to single-task performance and is calculated by dividing the difference in value (e.g., gait speed) between single and dual-task performance by the value of the single-task performance, expressed as percentage [23]. Negative DTE indicates that performance deteriorated in the dual-task condition relative to single-task performance (dual-task cost), while positive DTE represents improvement in performance (dual-task benefit). For the Stroop and clock tasks, we calculated a composite DTE for cognitive-task performance by summing the DTE for reaction time and accuracy, which accounts for speed-accuracy tradeoffs in the overall DTE [23]. We computed a composite DTE for the speech variables (clauses per utterance, pauses per utterance) in the same way.

Secondary outcome measures were the Timed Up and Go (TUG) test [24], the Activities-specific Balance Confidence (ABC) scale [25], and the Subjective Index of Physical and Social Outcome (SIPSO) [26]. The secondary outcome measures were assessed before and after intervention and at the one-month follow up assessment. These measures were collected immediately after the assessment of dual-task interference described above. The order of secondary outcome assessments was consistent across participants and timepoints (pre, post, follow-up). Additionally, at baseline, the participants were assessed on a range of measures to characterize severity of impairment in cognition (Montreal Cognitive Assessment [27]), executive function (Stroop color-word interference test [28]), language ability (Shipley Vocabulary Test [29]), lower extremity motor function (Fugl Meyer et al. [30]), and depression (short form of the Geriatric Depression Scale [31]). The 6-minute walk test was conducted to provide a measure of walking endurance.

Feasibility of DTGT was assessed by measuring participants' perceptions of physical and mental fatigue as well as their perceptions of task difficulty, anxiety, and fear of falling on a 100-mm visual analogue scale [12], where 0 represented no fatigue/anxiety/difficulty/fear, and 100 represented maximum levels of the construct. Adherence to the 12-session training program was a further measure of feasibility. Safety of the intervention was assessed via monitoring of adverse events and falls. Overall patient acceptance of DTGT was measured using a feedback questionnaire regarding the participants' satisfaction with the intervention.

## 6. Outcomes

All seven participants completed 12 sessions of DTGT within 6 weeks (range: 3.7–5.4 weeks, mean: 4.3). One patient missed the midpoint assessment due to scheduling difficulties, and two participants were not available for the follow-up assessment. Only two participants were assessed on the motor-motor dual-task combination. The reasons for not assessing other participants on the coin transfer task included inability to perform the task due to upper extremity hemiparesis or use of assistive device in the unaffected hand and insufficient time/minimizing testing fatigue. The baseline characteristics and demographics of the participants are presented in Table 4.

### 6.1. Gait Speed

Table 5 presents single and dual-task gait speeds for each participant at each assessment. Dual-task changes are shown in both absolute (gait speed, m/s) and relative (dual-task effects, %) measures. Baseline single-task gait speeds ranged from 0.58 m/s to 1.07 m/s (mean: 0.84 m/s). After intervention, single-task gait speed was generally maintained in all participants (mean: 0.87 m/s, range: 0.66–1.10). Of the five participants with follow-up data, three had further improvements in single-task gait speed at 1-month follow up. As illustrated in Table 5, all participants had dual-task declines in gait speed in all of the dual-task combinations at baseline, except for Participant 2 who essentially maintained single-task walking speed during the speech dual-task (+0.01 m/s). After intervention, five of the seven participants demonstrated a reduced dual-task cost in gait speed in at least one of the dual-task combinations. Participant 3, who demonstrated an increase in single-task gait speed post intervention, only improved his dual-task walking speed in the speech dual-task. Participant 6 walked faster in all conditions after intervention, but continued to experience dual-task costs on gait speed in all three cognitive-motor dual-tasks.

The effects of the intervention on absolute and relative dual-task costs on gait speed appeared to be different across dual-task combinations (Table 5). The most consistent improvements were observed for the Stroop task. At baseline, absolute dual-task declines in gait speed for the Stroop task ranged from 0.03 to 0.38 m/s (mean: 0.13 m/s). After intervention, four participants (P1, P2, P4,and P5) had noticeably smaller dual-task declines in gait speed (range for all participants: −0.01–0.17 m/s, mean: 0.06 m/s). The improvements in gait-related dual-task performance were less consistent and, on average, of smaller magnitude for the clock and speech tasks. Baseline dual-task declines in gait speed during the clock task ranged from 0.05 to 0.22 m/s (mean: 0.11 m/s); after intervention the dual-task declines were 0.00–0.16 m/s (mean: 0.09 m/s). For the speech task, baseline dual-task declines in gait speed ranged from −0.01 to 0.23 m/s (mean: 0.11 m/s); after intervention the dual-task declines were 0.02–0.31 m/s (mean: 0.14 m/s). The two participants who performed the coin transfer task slowed their gait speed at baseline by 0.20 m/s (Participant 1) and 0.12 m/s (Participant 4); after intervention the dual-task declines were 0.19 m/s (Participant 1) and 0.00 m/s (Participant 4).

The magnitude of the absolute dual-task decline in gait speed at baseline meant that three of the four participants with usual gait speed ≥0.80 m/s (P4, P5, P7) walked <0.80 m/s in at least one of the dual-task conditions. This is important, since 0.8 m/s is the widely considered threshold for functional community ambulation [22, 32]. Thus, the dual-task decline in these participants is clinically significant; they were unable to maintain gait speed needed for functional community ambulation when walking while performing a cognitive task. After intervention, all three of these participants had dual-task gait speeds >0.80 m/s for the Stroop and clock tasks, and two (P5 and P6) also walked >0.80 m/s during the speech task. These represent clinically meaningful improvements, since dual-task walking speeds have crossed into the range needed for full community ambulation [22, 32]. Of the three participants with single-task gait speed <0.80 m/s at baseline (i.e., functionally limited community ambulators during single-task walking), two (P1 and P2) had improved dual-task gait speeds in all dual-task conditions at the 1-month follow up, but remained <0.70 m/s. Participant 3 was the only participant to walk slower than 0.60 m/s at baseline. Although his gait speed increased to 0.66 m/s after intervention, this was not retained at follow up. Despite improved dual-task walking speed in the speech dual-task after intervention, his relative dual-task costs after intervention were worse than at baseline (Table 5).

### 6.2. Pattern of Cognitive-Motor Interference

**Figure 1:**
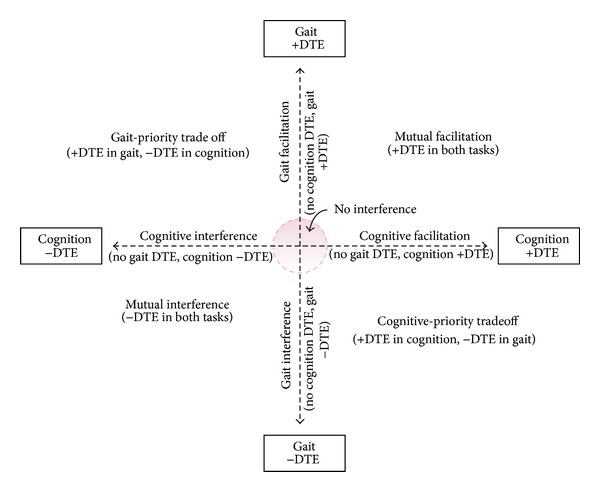
Patterns of cognitive-motor interference. Positive values for dual-task effects (DTE) indicate that performance improved in dual-task condition relative to single-task performance; negative values for DTE indicate that performance deteriorated in dual-task condition relative to single-task performance. Figure adapted from conceptual framework of Plummer et al. [9].

**Figure 2:**
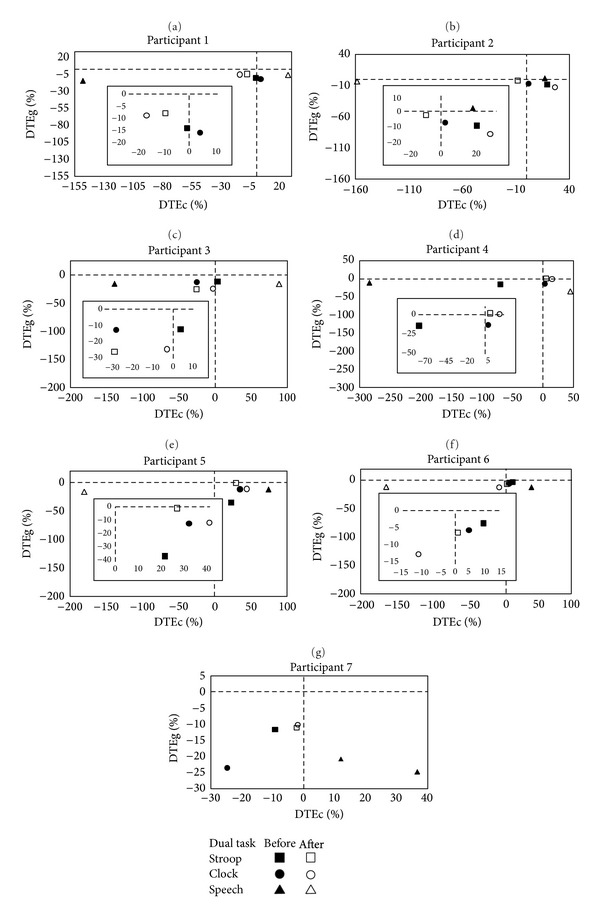
Plots showing patterns of cognitive-motor interference for each participant for each cognitive-motor dual-task combination before and after the intervention. Dual-task effects (DTE) represent percent change relative to single-task performance and are calculated by dividing the difference between single-task and dual-task values by the single-task value, expressed as a percentage. Positive values for DTE indicate that performance improved in the dual-task condition relative to single-task performance; negative values for DTE indicate that performance deteriorated in dual-task condition relative to single-task performance. DTEg is DTE on gait speed; DTEc is composite DTE for the three cognitive tasks (reaction time and accuracy for Stroop and clock tasks, clauses per utterance and pauses per utterance for spontaneous speech task).

To examine changes in the pattern of cognitive-motor interference, we plotted the DTE on gait speed (DTEg) against the DTE on cognitive-task performance (DTEc) for each subject for the three cognitive-motor dual-task combinations before and after the intervention.  Figure 1 explains the patterns of cognitive-motor interference, and Figure 2 presents the individual cognitive-motor interference patterns at baseline and postintervention assessments.

The data in Figure 2 indicate clearly the consistent negative DTE on gait speed (i.e., DTEg values consistently below the horizontal dotted line) and a range of positive and negative DTE in the cognitive task (i.e., DTEc values left and right of the vertical dotted line). Indeed, the most common patterns of cognitive-motor interference were* mutual interference* (dual-task costs for both tasks),* gait interference* (dual-task costs on gait with little change in cognitive task performance), and* cognitive-priority trade-off* (dual-task costs on gait with concurrent dual-task improvements in cognition). Another noteworthy observation of the data in Figure 2 is the variability in dual-task interference across tasks and between participants. The variability suggests that the way in which the participants allocated their attention between the simultaneous tasks changed across tasks and, in some participants, changed over time.

For example, Participant 1 demonstrated* gait interference* in the Stroop and clock dual-tasks at baseline, but* mutual interference* after intervention in both the Stroop and clock dual-tasks (Figure 2). Specifically, after the intervention, there were smaller dual-task costs on gait speed in both tasks, but this came at a cost to cognitive-task performance. One interpretation is that, at baseline, the participant prioritized his attention during dual-task walking on the cognitive task (illustrated by the absent/minimal DTEc), but after training he was able to divide attention between both tasks. His cognitive-motor interference pattern was different for the speech dual-task; he had* mutual interference* at baseline (with greater dual-task costs on speech than gait), but demonstrated* cognitive-priority trade-off* after the intervention (Figure 2). Despite the apparent trade-off of attentional resources during the speech dual-task after training, gait dual-task costs were smaller, suggesting an overall improvement in dual-task capacity in this task.

Summarizing the data illustrated in Figure 2, Participants 2, 4, 5, and 7 demonstrated improved dual-task capacity in the Stroop dual-task after intervention; their dual-task interference “point” for the Stroop dual-task was closer to the* no interference* region after intervention. Only Participants 4 and 7 showed similar postintervention improvements in the clock dual-task: Participant 4 had only* gait interference* at baseline, and after intervention he had* no interference* on gait and some improvement in clock-task performance (positive DTEc,* cognitive facilitation*); Participant 7 had* mutual interference* during the clock dual-task at baseline, but less* gait interference* and* no interference* in cognition after intervention. Participants 2, 3, and 6 were similar to Participant 1 described above in that they demonstrated a change in pattern of interference for the clock dual-task, suggestive of a change in spontaneous prioritization of attention after intervention. However, the pattern change was not consistent (see Figure 2).

DTE on speech measures was typically much larger than those for the Stroop and clock task measures. The participants were also highly variable in whether they had negative or positive dual-task effects on measures of speech performance, and the pattern changed after intervention in most participants. For example, in Participants 1, 3, and 4, the pattern of dual-task interference in the speech dual-task changed from* mutual interference* to* cognitive-priority trade-off*; whereas in Participants 5 and 6, the patterns changed from* cognitive-priority trade-off* to* mutual interference* (in both cases, DTEg did not change but DTEc shifted from positive to negative).

### 6.3. Balance and Participation

Five of the seven participants had increased balance confidence (ABC scores) after intervention (Table 6), but only Participants 2 and 6 had increases that exceed the standard error of measurement (6.81 points) for the ABC in chronic stroke [33]. Compared to only two participants at baseline, four of the five participants tested 1 month after the intervention had ABC scores >80%, indicative of high physical functioning [34] and low likelihood of recurrent falls [35]. Participant 3 was low functioning at baseline (ABC < 50%); this improved slightly after intervention, but was worse than baseline at follow up. The reason why Participant 5 scored so low on the ABC scale after intervention is unclear. Three participants had increased SIPSO scores after intervention, suggestive of higher levels of social and physical reintegration, but the minimal clinically important difference values for this measure are not known. There were no meaningful changes in the TUG after intervention; the only participant whose pre-post change in TUG exceeded the minimal detectable change value for chronic stroke (2.9 seconds) [36] was Participant 3, whose baseline TUG indicated high fall risk [37]. He showed decline in functional mobility after training (4.3 seconds slower). Indeed, Participant 3 disclosed during the postintervention evaluation that he had experienced a fall (with associated vertigo) at home since the midpoint assessment.

### 6.4. Feasibility and Safety Outcomes

Most participants reported small increases in mental and physical fatigue in each session, with the ratings of physical fatigue typically greater than those of mental fatigue. The participants also reported low levels of difficulty, anxiety, and fear of falling. Participant 3 had the highest average ratings of perceived difficulty (mean: 55.3 mm, range: 34–68), anxiety (mean: 55.0 mm, range: 37–70), and fear of falling (mean: 47.5 mm, range: 28–72). None of the participants missed any session. Other than Participant 3 who reported a fall without injury at home during the second half of the intervention period, there were no adverse events reported. All participants indicated that they were “extremely satisfied” (*n* = 4) or “satisfied” (*n* = 3) with the treatment.

## 7. Discussion

This case series is the first study to examine cognitive-motor dual-task gait training in community-dwelling adults with stroke. The findings from this report provide evidence that cognitive-motor dual-task gait training is feasible in the first 12 months after stroke, and that cognitive-motor dual-task gait training can improve dual-task walking speed. The most consistent improvements were observed for the Stoop dual-task; improvements in dual-task walking speed during the clock and speech tasks were more variable. This may indicate low transfer of training to untrained cognitive-motor dual-tasks. The cognitive tasks used in the DTGT intervention largely involved executive functioning (which is a key function required for the Stroop task), thus it is possible that improvements in the Stroop dual-task were greater than the clock and speech dual-tasks because it resembled most closely the types of cognitive activities used during DTGT. Another possible explanation for the smaller and less consistent changes in dual-task performance in the clock and speech task, however, is that these tasks were relatively more difficult than the Stroop task. Previous research has shown significantly greater dual-task effects on gait in the clock [38] and speech tasks [8, 39] in people with stroke and healthy adults. Regardless of whether this finding can be attributed to poor transfer of training to untrained dual-task combinations, or differences in cognitive-task difficulty, the practical implication is that it may be important to include a wider range of cognitive tasks during DTGT to maximize transfer to different cognitive-motor dual-task combinations. Alternatively, therapists may choose to assess dual-task performance in a range of different dual-task combinations and then select dual-task activities for intervention that specifically target the person's greatest limitations.

An interesting finding from this case series is that most participants did not demonstrate remarkable improvements in single-task walking speed after the intervention. This may be due to the fact that there was no single-task practice during the DTGT sessions; the training focused exclusively on dual-task practice of gait. If gains in single-task walking speed are an important therapy goal, then it may be necessary to include both single and dual-task practice components in a dual-task gait training program. This may be a worthy consideration for future DTGT interventions, since single-task gait speed has been found to be correlated with dual-task costs on gait speed [39, 40]. Nevertheless, most of the participants in this case series showed improvements in dual-task walking in at least one of the dual-tasks.

Despite the improvements in the absolute and relative dual-task effects on gait speed by most of the participants in this study, our analysis of the patterns of cognitive-motor interference showed tremendous variability in dual-task performance and in the effects of the intervention on dual-task walking. While some participants showed evidence of improved dual-task capacity (e.g., decreased dual-task costs in both tasks), others demonstrated differences in the way they performed the task with no clear evidence of improvement in dual-task capacity. It is unclear whether a change in the pattern of cognitive-motor interference is indicative of a change in dual-task ability, and unfortunately this cannot be elucidated from the current data. However, our unique analysis highlights several important factors to be considered in future studies of dual-task gait training interventions. First, analysis of dual-task effects on gait may be inadequate to assess changes in dual-task performance. Performance on both gait and nongait tasks must be analyzed, and changes relative to each other should be considered when interpreting effects of treatment on dual-task performance. Second, reliability of dual-task performance needs to be established. To date, there have been no studies that have examined the reliability of dual-task performance: do individuals perform a particular dual-task the same way on multiple occasions? Until this is determined, it is not possible to conclude that changes in the pattern of interference are due to the intervention or just to a different strategy on a different day. This raises a third issue: the influence of instructions on dual-task performance. We did not provide any specific instruction about which task the individual should prioritize during the dual-task assessments. We were interested in examining the “default” performance of the participants. Lack of specific instruction about which task to prioritize means that each individual shall decide how to prioritize their attention in a given dual-task situation. While it is reasonable to assume that dual-task practice (i.e., dual-task intervention) may change the way a person chooses to spontaneously allocate their attention during dual-task walking, there are several other factors that may influence attention allocation in a particular dual-task situation, such as the perceived difficulty or importance of one task over another. Does dual-task training change a person's preferred attention allocation strategy or improve the efficiency and/or capacity for attention shifting, or simply improve gait automaticity such that attention can now be more easily allocated to other tasks?

Recent research has suggested that the ability to flexibly allocate attention between the two tasks during dual-task walking may be an important factor influencing dual-task interference [41]. It is not yet known whether individuals with stroke have the ability to voluntarily adapt their attentional focus during dual-task walking. While our results demonstrate variability within and between individuals in the way attention may be prioritized in different dual-tasks, determining the influence of dynamic attentional prioritization on dual-task performance will be important in order to establish the optimal instructions to use in dual-task assessment and training after stroke.

A potentially important clinical implication of the observations from this case series concerns the appropriateness of DTGT for individuals with more severe balance and gait deficits and/or limitations in cognitive ability. Participant 3 was the slowest walker and was at high risk for falls, with a baseline TUG greater than 15 seconds [37], and he had the lowest score on the MoCA. Relative to baseline, this participant had greater dual-task costs on gait speed in all three cognitive-motor dual-tasks after intervention, as well as clinically significant decline in the TUG. Furthermore, he perceived the intervention to be relatively difficult with some associated anxiety and fear of falling. It is possible that for individuals with considerable gait and balance impairments, improvements in walking and balance in undistracted conditions are needed before DTGT can be effectively implemented; or it may be that DTGT is not appropriate for individuals with a combination of physical and cognitive impairments. The findings from this case series have informed the selection criteria in the follow-up randomized controlled trial of dual-task gait training versus single-task gait training in community-dwelling stroke survivors within one year after stroke [42].

A limitation of this case series is that we did not include any postintervention assessments of cognition. In future, it would be of interest to determine whether DTGT has any effect on cognitive domains, such as executive function. The nature of the case series also limits the generalizability of the findings to other individuals with stroke. An unexpected scenario was that all of the participants in this case series were male. This is important because gender differences in gait-related dual-task interference have been reported among healthy adults [43, 44]. In general, there were only small changes in the self-reported measure of participation used in this study. Examination of spontaneous physical activity using activity monitoring devices may yield more accurate information about whether improvements in dual-task walking are translated to increased community ambulation. One of the participants (P5) in this case series for whom we captured physical activity data using the PAMSys (Biosensics, Cambridge, MA; data not shown) demonstrated notable increases in walking and standing activity, number of episodes of walking per day, and number of steps per day after the intervention [45].

## 8. Conclusion

The findings of this case series demonstrate the feasibility of DTGT in community-dwelling stroke survivors within one year of stroke. Our observations suggest that the potential benefits of DTGT may be limited in individuals with poor balance, slow usual walking speed, and/or impaired cognitive ability. The type and variety of cognitive tasks used during DTGT may influence transfer to untrained dual-task combinations. Importantly, even though dual-task costs on gait speed may improve with DTGT, the pattern of DTE suggests that changes in overall dual-task performance with intervention are highly variable.

## Figures and Tables

**Table 1:** Overview of gait activities for dual-task gait training.

	Predictable	Unpredictable
Stationary	Closed tasks (i) Walking in flat, wide space (ii) Walking over/around obstacles, all of the same height and equally spaced (iii) Walking with narrow BOS	Variable motionless tasks (i) Walking around obstacles with variable spacing (ii) Walking over obstacles of variable height (iii) Walking over changing floor surfaces

Moving	Consistent motion tasks (i) Walk toward/beside/behind a person moving at a consistent speed and direction (ii) Walk under different lighting conditions	Open tasks (i) Walking in a crowded corridor (ii) Walking outdoors in the car park (iii) Walking and negotiating a moving obstacle

**Table 2:** Summary of cognitive tasks for dual-task gait training.

Task	Description
Random number/letter generation	(i) Randomly naming numbers between 100 and 500 (without repetition or consecutively) (ii) Randomly naming odd (or even) numbers between 1 and 100 (without repetition or consecutively) (iii) Randomly naming consonants of the alphabet (without repetition or consecutively)

Word association	Easy: (i) naming as many words as possible in a category (e.g., animals, fruits) (ii) naming opposites of words Hard: (i) naming as many words as possible beginning with a particular letter (ii) naming as many words as possible in a category (e.g., European cities)

Working memory	Easy: (i) reciting a sequence of numbers (3 or 4 number sequences) (ii) reciting grocery list (3-4 items) Hard: (i) reciting a sequence of numbers (5 numbers per sequence) (ii) reciting grocery items (5 items)

Calculating a time	Easy: adding or subtracting minutes to a given time within the hour (e.g., 3:15 + 5 minutes; 1:30 − 15 minutes) Hard: adding or subtracting minutes to a given time into the next hour (e.g., 4:40 + 25 minutes; 1:15 − 30 minutes)

Backward recitation	(i) Reciting number sequences backward (ii) Months of the year (iii) Days of the week (iv) Backward spelling (4-5 letter words) (v) Counting backward (by 2, 3, 6, 7, and 8; starting between 75 and 100)

**Table 3:** Dual-task assessments.

Task	Description	Transfer
Stroop task	An executive function task representing the type of tasks trained during practice. Participants hear the words “high” and “low,” spoken in either a high pitch or a low pitch; participants are instructed to report the pitch of the word (high/low), ignoring the actual word	Trained

Clock task	A visuospatial cognition task. Participants are instructed to generate a mental representation of a clock face and respond verbally (yes/no) based on where the hands of the clock would be for the given times	Untrained

Spontaneous speech	Spontaneous narrative in response to a stimulus question; highly relevant to everyday dual tasking	Untrained

Coin-transfer task	Participants wear a belt with pockets and transfer coins from one pocket to another	Untrained

**Table 4:** Demographic and baseline characteristics of participants.

Participant	Age (years)	Gender	Time since stroke (months)	Side of hemiplegia	Fugl-Meyer	MoCA	Stroop test	Shipley	GDS	Education (years)	6 min walk (m)	Assistive device
1	72	M	12	L	24	27	39	32	3	16	243.2	None
2	74	M	11	R	25	25	49	31	3	16	230.9	Cane
3	86	M	4.5	L	26	23	NT	33	3	16	198.7	Cane
4	42	M	11	L	25	26	26	27	8	12	318.9	None
5	76	M	8.5	L	29	28	30	37	0	22	373.8	None
6	60	M	9.3	L	27	28	22	33	3	18	358.8	Cane
7	86	M	3	R	26	27	45	37	2	22	324.5	None

Mean	70.9		8.5		26.0	26.3	35.2	32.9	3.1	17.4	292.7	
SD	15.5		3.5		1.6	1.8	10.8	3.5	2.4	3.6	68.0	

Fugl-Meyer: Fugl-Meyer motor assessment for lower extremity (max. 34); MoCA: montreal cognitive assessment (maximum score 30); Stroop test: Stroop color-word interference score (color-word score minus color score); Shipley: Shipley vocabulary test (max. 40); GDS: geriatric depression scale (score > 5 indicates depression); NT: not tested.

**Table 5:** Absolute (gait speed, m/s) changes in gait speed for each participant at pre, mid, post intervention and 1-month follow up; and relative (dual-task effects, %) pre and post intervention. NT indicates not tested.

	Single task	Dual Stroop		Dual clock		Dual speech		Dual coin	
Participant 1									
Pre	0.77	0.67	(−13.7%)	0.66	(−14.8%)	0.64	(−17.0%)	0.57	(−26.1%)
Mid	0.74	0.69		0.72		0.66		0.65	
Post	0.75	0.69	(−7.3%)	0.69	(−8.1%)	0.68	(−8.8%)	0.58	(−22.6%)
Follow up	0.83	0.79		0.71		0.69		0.69	
Participant 2									
Pre	0.68	0.62	(−8.2%)	0.64	(−6.6%)	0.69	(+1.8%)	NT	
Mid	0.77	0.78		0.75		0.71			
Post	0.70	0.69	(−2.3%)	0.61	(−13.0%)	0.68	(−3.3%)		
Follow up	0.83	0.75		0.68		0.76			
Participant 3									
Pre	0.58	0.51	(−12.2%)	0.51	(−12.6%)	0.49	(−15.7%)	NT	
Mid	0.61	0.62		0.52		0.52			
Post	0.66	0.49	(−25.8%)	0.50	(−24.3%)	0.56	(−16.0%)		
Follow up	0.57	0.55		0.51		0.56			
Participant 4									
Pre	0.90	0.77	(−14.2%)	0.78	(−13.1%)	0.81	(−10.1%)	0.78	(−12.8%)
Mid	0.75	0.73		0.73		0.69		0.65	
Post	0.92	0.93	(+1.4%)	0.92	(0)	0.61	(−33.9%)	0.92	(0)
Follow up	1.03	1.04		0.96		0.76		0.98	
Participant 5									
Pre	1.07	0.68	(−36.0%)	0.94	(−12.1%)	0.94	(−12.3%)	NT	
Mid	0.99	0.86		0.91		0.84			
Post	1.02	1.01	(−1.0%)	0.90	(−11.4%)	0.86	(−16.1%)		
Follow up	NT	NT		NT		NT			
Participant 6									
Pre	0.97	0.93	(−3.5%)	0.91	(−5.5%)	0.85	(−12.2%)	NT	
Mid	1.03	0.94		0.90		0.87			
Post	1.11	1.05	(−6.2%)	0.98	(−12.2.%)	0.98	(−12.1%)		
Follow up	1.05	1.02		1.01		0.89			
Participant 7									
Pre	0.92	0.81	(−11.7%)	0.70	(−23.5%)	0.69	(−24.7%)	NT	
Mid	NT	NT		NT		NT			
Post	0.90	0.80	(−11.1%)	0.81	(−10.1%)	0.71	(−20.8%)		
Follow up	NT	NT		NT		NT			
Mean (SD)									
Pre	**0.84** (0.17)	**0.71** (0.14)	**−14.1%** (10.3)	**0.73** (0.15)	**−12.6%** (5.9)	**0.73** (0.15)	**−12.9%** (8.0)		
Post	**0.87** (0.17)	**0.81** (0.20)	**−7.5%** (9.1)	**0.77** (0.18)	**−11.3%** (7.3)	**0.72** (0.15)	**−15.8%** (9.8)		

**Table 6:** Secondary outcomes pre and, post training and, if possible, at one month follow up.

	Timed up and go (s)			ABC Scale (%)			SIPSO (max. 40)		
	Pre	Post	Follow up	Pre	Post	Follow up	Pre	Post	Follow up
Participant 1	14.4	13.8	14.6	88.6	92.5	94.4	33	27	32
Participant 2	13.5	14.3	13.5	60.6	73.8	83.1	25	30	33
Participant 3	15.0	19.3	20.1	48.8	53.1	34.4	22	23	22
Participant 4	11.3	9.0	10.2	66.9	71.9	85	14	19	25
Participant 5	11.5	11.5	NT	73.1	46.3	NT	26	32	NT
Participant 6	10.8	9.7	10.2	75	91.6	88.8	31	33	33
Participant 7	10.5	10.5	NT	81.9	81.9	NT	34	33	NT

Mean	12.4	12.5		70.7	73.0		26.4	28.1	
Median	11.5	11.5		73.1	73.8		26	30	

ABC: activities-specific balance confidence scale, SIPSO: subjective index of physical and social outcome, NT: not tested.
